# Spores of arbuscular mycorrhizal fungi inhabiting the insides of mossballs formed by *Rigodium implexum*

**DOI:** 10.17912/micropub.biology.001982

**Published:** 2026-05-06

**Authors:** Roberto Godoy, Paula Aguilera, Javier Retamal, Erwin Guzmán Riffo, Lara J. Fox, Ava M. Cooper, Constanza Martínez Manzano, César Marín

**Affiliations:** 1 Instituto Ciencias Ambientales y Evolutivas, Austral University of Chile, Valdivia, LR, CL; 2 Departamento de Ciencias Agropecuarias y Acuícolas, Temuco Catholic University, Temuco, AR, CL; 3 Departamento de Investigación e Innovación, spin off Universitaria Myconativa; 4 Centro de Investigación e Innovación para el Cambio Climático (CiiCC), Universidad Santo Tomás; 5 Durham University, Durham, ENG, GB; 6 Escuela de Ingeniería en Conservación de Recursos Naturales, Austral University of Chile, Valdivia, LR, CL; 7 Amsterdam Institute for Life and Environment, Vrije Universiteit Amsterdam

## Abstract

The spores of four species of arbuscular mycorrhizal fungi (Phylum: Glomeromycota): *Acaulospora laevis* , *Acaulospora sieverdingii* , *Ambispora gerdemannii* , and *Dominikia aurea* , were found inhabiting the insides of mossballs formed by *Rigodium implexum* at two forest sites in southern Chile. These species were identified through morphological keys. *R. implexum* mossballs are usually 10-20 cm in diameter, unattached, and globose, and are found in large masses on the floor of Valdivian temperate rainforests. This phenomenon is reported for the first time, and possible co-dispersion mechanisms require further research. A methodology to extract Glomeromycota spores from this type of plant material is also presented.

**Figure 1. Glomeromycota spores inhabiting the insides (photos shown in C to F) of Rigodium implexum mossballs:** **A.** Forest floor covered by *R. implexum* at San Martín Research Forest (SMRF), Los Ríos Region, Chile. **B.** Close-up of mossballs formed by *R. implexum* . **C.** *Acaulospora laevis* (SMRF). **D.** *Acaulospora sieverdingii* (SMRF). **E.** *Ambispora gerdemannii* (SMRF) *.* **F.** *Dominikia aurea* (near Ranco Lake coast, Los Ríos Region, Chile).

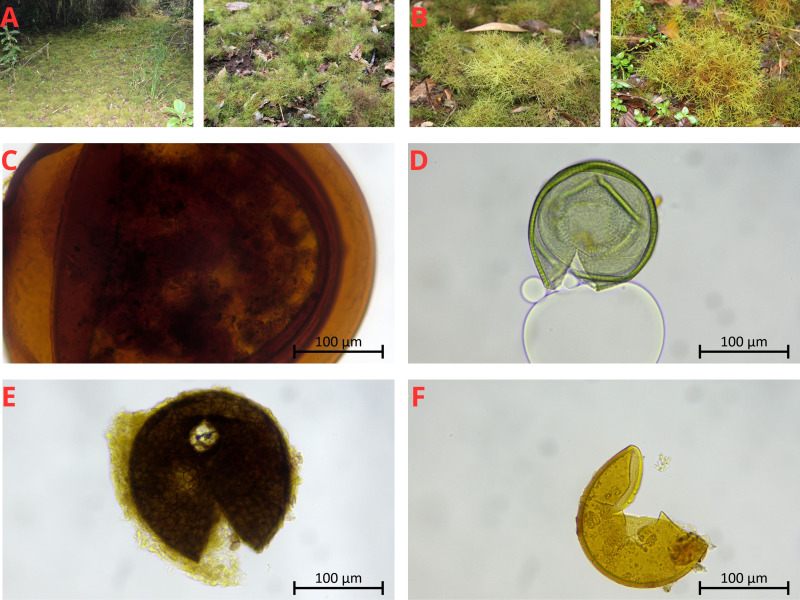



## Description

Spores of four species ( *Acaulospora laevis, Acaulospora sieverdingii, Ambispora gerdemannii,* and *Dominikia aurea* ) of arbuscular mycorrhizal fungi (AMF) (Phylum: Glomeromycota) were found inhabiting the insides of mossballs formed by *Rigodium implexum* ( **Fig. 1** ), which grow on the forest floor of the Valdivian rainforests of Chile (Villagrán Moraga, 2000; Frahm, 2001).

Mossballs formed by *R. implexum* are a unique phenomenon. Although in southern Chile, forest floors covered with *R. implexum* mossballs were first mentioned by Herzog (1939) and reported in detail by Frahm (2001), little research has been conducted on this system. These mossballs are usually 10-20 cm in diameter, unattached, and globose, found in large masses on the forest floor, accumulating in shallow forest depressions or on flat land, where they are sometimes scattered by wind (Frahm, 2001). Each one of these mossballs constitutes a single densely branched plant, whose stiff, scale-leaved branches maintain the globular shape whether wet or dry (Frahm, 2001). *R. implexum* is distributed at low elevations (<400 m) of the Valdivian temperate rainforest (mainly in Chile), and mostly in protected areas (Villagrán Moraga, 2000; Frahm, 2001). There are many unknown aspects of the biology of these mossballs, for example, regarding the mechanisms of their propagation (i.e., by fragmentation, wind movement, and/or birds), nutrient acquisition strategies (i.e., by atmospheric inputs and/or periodic floods), and their habitat dynamics (i.e., moisture regime, micro-ecology) (Frahm, 2001).

Arbuscular mycorrhizal fungi (AMF) are among the oldest symbioses on Earth, with fossil evidence dating back approximately 407 million years (Strullu-Derrien et al., 2018). These fungi are estimated to associate with around 72% of global land plants (Meng et al., 2023). In this symbiosis, plants allocate photosynthetically derived carbon in exchange for nutrients such as phosphorus and nitrogen, while also benefiting from enhanced tolerance to biotic and abiotic stresses (Smith and Read, 2008). AMF are traditionally considered obligate, clonal symbionts that reproduce asexually via spores, which vary widely in traits such as volume, ornamentation, investment, shape, and color (Pehim Limbu et al., 2025). AMF spores seem to be mainly dispersed through wind and animals (Paz et al., 2021), while co-dispersion mechanisms with plants, particularly in South America, remain unexplored (Paz et al., 2021). Similarly, associations between mosses and Glomeromycota remain understudied (Pressel et al., 2021). Under this context, investigating AMF spores inhabiting the insides of mossballs formed by *R. implexum* fills several knowledge gaps.

We extracted AMF spores from three sample types across southern Chile: mossballs in San Martín Research Forest (SMRF) and near Ranco Lake' coast, and bulk soil surrounding mossballs at SMRF. Two of the four reported species were found in mossballs in SMRF: *Ac. laevis* and *D. aurea* , while all four species were found in the surrounding bulk soil. Three of the four species were found at Ranco Lake coast' mossballs: *Ac. laevis* , *Ac. sieverdingii* , and *Am. gerdemannii* . As expected, AMF spores in bulk soil were more easily extracted, detected, and abundant (at least one order of magnitude). In contrast, within *R. implexum* mossballs, they were more scarce and difficult to extract. All four AMF species have been previously reported in Chile (Marín et al., 2017, 2025); *Ac. laevis* is particularly common and abundant across the country (59 occurrences according to the Global Biodiversity Information Facility – GBIF: Marín et al., 2025). Two of the four AMF species ( *Ac. laevis* and *D. aurea* ) were found in all three samples, despite the two sites (SMRF and Ranco Lake' coast) being 98 km apart. This is not surprising as Glomeromycota is characterized, in general, by low levels of endemism: globally, a third of AMF taxa are present in all five continents (Davison et al., 2015), while in Chile, a third of AMF species are shared between pristine forest ecosystems and agroecosystems (Marín et al., 2017). In a recent survey of AMF morphological biodiversity across 34 vineyards distributed along a 1,000 km climatic gradient across Chile, from Coquimbo (29° 54ʹ S) to La Araucanía (38° 44ʹ S) administrative regions, a total of 15 AMF species and more than 94,000 spores were identified (Aguilera et al., 2024). From those 15 AMF species, *Ac. laevis* was present and dominant across the whole gradient, while *Am. gerdemannii* was also present across the gradient (albeit less abundantly), and *D. aurea* was found in a few vineyards. From the four AMF species, *Ac. laevis* was described first (Gerdemann and Trappe, 1974), while *Ac. sieverdingii* was described last (Oehl et al., 2011). *Ac. sieverdingii* was initially described from lowland temperate Europe and tropical West Africa (Oehl et al., 2011), something not rare in Glomeromycota, as for example, *Ambispora reticulata* (not reported in this study) was initially described from mountainous areas in Switzerland and Chile (Oehl et al., 2012). The AMF species *D. aurea* was previously known as ‘ *Glomus aureum* ’ (Oehl et al., 2003a; Błaszkowski et al., 2021, 2025).

Regarding the AMF spores found inside *R. implexum* mossballs, many questions remain: are these AMF species similar or different from the ones actually associating symbiotically with *R. implexum* ? Globally (Meng et al., 2023) and regionally (Godoy and Marín, 2019; Catania et al., 2025), many moss species are known to associate with AMF, but more research is needed in this field. How do the AMF spores actually get inside these mossballs? For how long and how far away could they travel on them? Are there co-dispersion processes going on? All of these are unanswered questions that should be addressed in future research. Current research on how AMF colonize and inhabit leaf litter (Bunn et al., 2019; de Lima et al., 2025) and how they are dispersed by wind via spore traits (Chaudhary et al., 2020; Pehim Limbu et al., 2025) could help solve these questions.

## Methods

In August 2025, mossballs formed by *Rigodium implexum* were collected from two sites in south-central Chile: Ranco Lake coast, Los Ríos Region, Chile (coordinates: -40.167987, -72.27136; 72 m.a.s.l.) and the San Martín Research Forest, which belongs to the Austral University of Chile, Los Ríos Region, Chile (coordinates: -39.648386, -73.195237; 98 m.a.s.l.). In the field, we collected superficial mossballs that were not in direct contact with the soil or other plants. The exterior of each mossball was dried up and cleaned with a paper towel to exclude environmental arbuscular mycorrhizal fungi (AMF) spores.

At each site, three replicates (three mossballs) were collected (each consisting of approximately 500 g of fresh plant material), placed in paper bags, and transported to the laboratory, where they were dried at room temperature for 24 hours. In the lab, the plant material was manually sectioned with scissors. From each replicate, a total of 20 g of dried material was weighed and placed in a 200 ml glass beaker. A volume of 100 ml of tap water was added, and the sample was then placed on a magnetic stirrer for 5 minutes to homogenize the sample and release the AMF spores from the plant material. Next, the solution was slowly poured through a series of sieves arranged in descending order of mesh size (1000, 500, 250, 106, 53, and 38 µm). The material retained on the 1000- and 500-µm sieves was discarded. The material retained on the 250, 106, 53, and 38 µm sieves was carefully rinsed with running water using a wash bottle to loosen any adhering spores and concentrate the material in one section of the 38 µm sieve. This was then transferred to Falcon tubes until a total volume of 25 ml was reached, and the tubes were placed in a test tube rack. Then, using a syringe with an extension tube inserted to the bottom of each Falcon tube containing the aqueous sample, a 70% sucrose solution was added until a final total volume of 50 ml was reached. The samples were brought to a constant weight and centrifuged at 3000 rpm for 10 minutes.

Subsequently, the supernatant from each sample was filtered through a 38 µm sieve, and the retained material was gently washed with running water to remove any remaining sucrose and avoid stressing and destroying spores. The contents of the sieve were poured into a flat-bottomed funnel lined with Whatman 2 filter paper, and filtration was facilitated using a vacuum pump. The material retained on the filter paper was then carefully transferred with spatula-tipped forceps to a labeled Petri dish for subsequent observation and selection of AMF spores under a stereomicroscope. The selected spores were transferred to glass slides using a dissecting needle for fixation in polyvinyl alcohol-glycerol lactic acid (PVLG) medium mixed 1:1 (v/v) with Melzer's reagent (Sieverding et al., 1991; Oehl et al., 2003b) for taxonomic identification. Spores were classified and taxonomically identified using classical morphological criteria and specialized taxonomic keys for Glomeromycota, based on Sieverding et al. (1991), Redecker et al. (2013), Oehl et al. (2003b, 2011a, 2011b), and Błaszkowski (2012). The traits considered for taxonomic identification included: spore size and color, wall structure, layers, ornamentation, and type of subtending hypha.
